# An unusual presentation of cutaneous pseudolymphoma^☆^

**DOI:** 10.1016/j.abd.2020.11.019

**Published:** 2022-07-16

**Authors:** Ying Wang, Sitong Li, Yanping Bai, Zhancai Zheng

**Affiliations:** Department of Dermatology, China-Japan Friendship Hospital, Beijing, China

Dear Editor,

Cutaneous Pseudolymphoma (CPL) refers to reactive lymphoid proliferation simulating cutaneous lymphomas. CPL may occur in response to many kinds of foreign antigens or factors, such as injected substances, tattoos, arthropod bites, and so on.^1^ However, in many cases, the reasons cannot be identified, hence the term idiopathic CPL. CPL has various clinical presentations, usually including red plaques, papules, and nodules. Herein, we report a case of idiopathic CPL with subcutaneous nodules on the back.

A 31-year-old man presented with a 3-month history of two asymptomatic subcutaneous nodules on his back. He was otherwise healthy and there was no history of preceding illness, injected substances, vaccination, or insect bite. Physical examination revealed two coin-sized subcutaneous nodules palpable on his back (Fig. 1). The skin overlying the nodules was normal. There was no lymphadenopathy or hepatosplenomegaly. The supposed clinical diagnosis of the nodules was lipoma before the biopsy. Tests for HBsAg, anti-HCV antibody, anti-HIV antibody, and syphilis antibody were negative. Chest, abdominal and pelvic CT did not reveal any abnormality. The biopsy specimen taken from a subcutaneous nodule showed lymphocytic nodular infiltrate with several reactive germinal centers, extending into subcutaneous fat (Fig. 2a). Some nuclei between the follicles were large and mildly irregular (Fig. 2b). Immunohistochemistry demonstrated positive for CD3, CD4, CD8, CD20, CD138, KAPPA (few and scattered), LAMBDA (few and scattered), Ki67 (presented a level proliferation index of about 15%), and negative for CD30. CD21 expression exhibited atrophic follicular dendritic cell network (Fig. 3). Polymerase chain reaction amplification showed polyclonality for immunoglobulin heavy chain and T-cell gamma chain gene rearrangements. Based on the above findings, a diagnosis of idiopathic CPL was rendered. The patient received surgical therapy. In the following-up seven years, the lesions did not reappear, and the patient was healthy.

**Figure 1:**
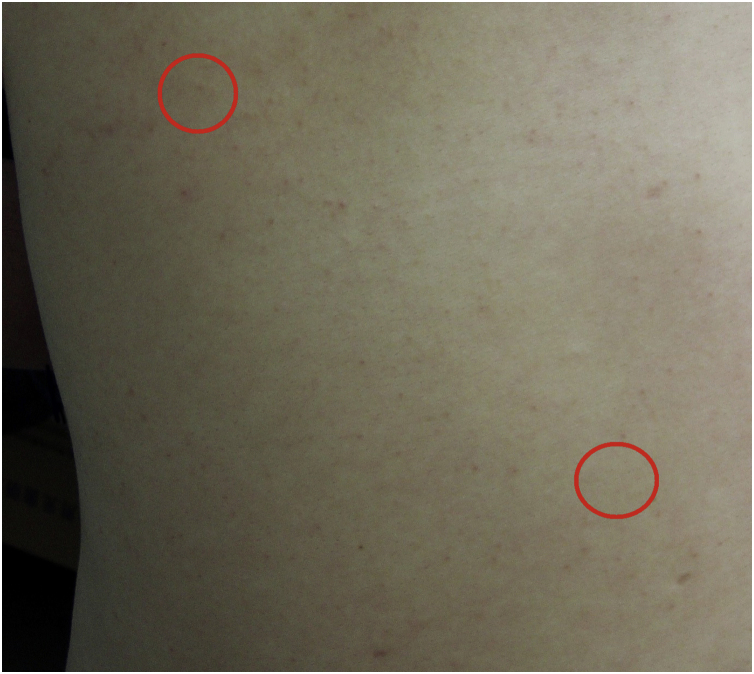
Clinical features at presentation. Two coin-sized subcutaneous nodules on the back (red circle), and the skin overlying the nodules was normal.

**Figure 2:**
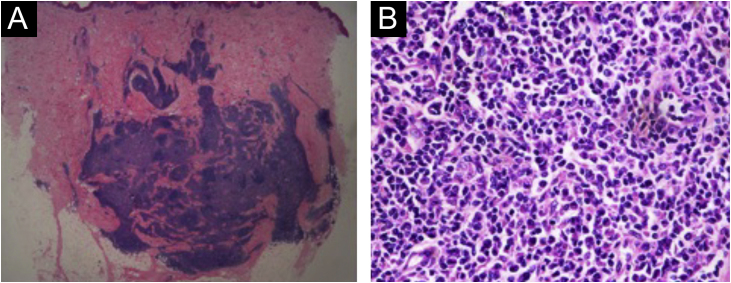
(A), lymphocytic nodular infiltrate with several reactive germinal centers extending into subcutaneous fat (Hematoxylin & eosin, ×10). (B), Some large, mildly irregular nucleus between the follicles (Hematoxylin & eosin, ×400).

**Figure 3:**
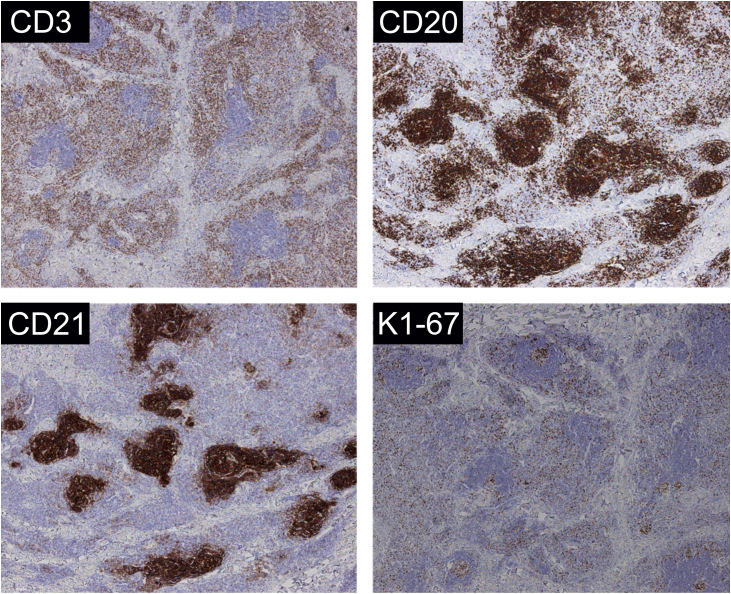
Immunohistochemistry of CD3, CD20, CD21, Ki67 (about 15%) were positive (original magnification ×40).

Cutaneous Pseudolymphoma (CPL) is not an uncommon condition, which considers a group of benign cutaneous lymphoproliferative disorders and very rarely progresses to lymphoma. The clinical presentation of CPL has a wide spectrum. The most common clinical manifestations are red to violaceous nodules, papules, or plaques on the exposed areas, especially on the face and neck. Subcutaneous nodules, as in our case, are the uncommon presentation of CPL, which have been described in several cases occurring secondary to feline scratches or injection of vaccines.^2^, ^3^, ^4^ In addition, the lesions in previous cases are all on extremities, especially upper arms. However, an etiology cannot be identified in our case, and the subcutaneous nodules are on the back. To our knowledge, this is the first report of idiopathic CPL with subcutaneous nodules on the back. CPL may resolve spontaneously or persist indefinitely. There are no specific treatments for CPL. Present therapeutic approaches include surgical excision, photodynamic therapy, interferon, radiotherapy, topical corticosteroids, and so on. Despite a relatively good prognosis, a few CPL can progress to lymphoma,^5^ so a long-term follow-up is indispensable.

## Financial support

None declared.

## Authors’s contributions

Ying Wang: Approval of final version of the manuscript; conception and planning of the study; drafting and editing of the manuscript.

Sitong Li: Approval of final version of the manuscript; participation in the design of the study.

Yanping Bai: Approval of final version of the manuscript; conception and planning of the study.

Zhancai Zheng: Approval of final version of the manuscript; conception and planning of the study; drafting and editing of the manuscript; critical review of the literature; critical review of the manuscript.

## Conflicts of interest

None declared.
